# The Health Impact of Social Community Enterprises in Vulnerable Neighborhoods: Protocol for a Mixed Methods Study

**DOI:** 10.2196/37966

**Published:** 2022-06-22

**Authors:** Erik Hendriks, Maria Koelen, Kirsten Verkooijen, Jan Hassink, Lenneke Vaandrager

**Affiliations:** 1 Health and Society Department of Social Sciences Wageningen University & Research Wageningen Netherlands; 2 Wageningen Plant Research Agrosystems Research Wageningen Netherlands

**Keywords:** social community enterprise, health, well-being, public health, social determinants of health, health inequalities, assets-based approach, conceptual modeling

## Abstract

**Background:**

This 4-year research project focuses on 6 social community enterprises (SCEs) that operate in 5 neighborhoods in a Dutch city. Residents of these neighborhoods face problems such as poor average levels of physical and mental health, high unemployment rates, and weak social cohesion. SCEs offer residents social, cultural, and work-related activities and are therefore believed to help these persons develop themselves and strengthen the social ties in the community. Because of a lack of empirical evidence; however, it is unclear whether and how SCEs benefit the health and well-being of participants.

**Objective:**

This paper outlines a protocol for an evaluation study on the impact of SCEs, aiming to determine (1) to what extent SCEs affect health and well-being of participating residents, (2) what underlying processes and mechanisms can explain such impact, and (3) what assets are available to SCEs and how they can successfully mobilize these assets.

**Methods:**

A mixed methods multiple-case study design including repeated measurements will be conducted. Six SCEs form the cases. An integrated model of SCE health intervention will be used as the theoretical basis. First, the impact of SCEs is measured on the individual and community level, using questionnaires and in-depth interviews conducted with participants. Second, the research focuses on the underlying processes and mechanisms and the organizational and sociopolitical factors that influence the success or failure of these enterprises in affecting the health and well-being of residents. At this organizational level, in-depth interviews are completed with SCE initiators and stakeholders, such as municipal district managers. Finally, structurally documented observations are made on the organizational and sociopolitical context of the SCEs.

**Results:**

This research project received funding from the Netherlands Organization for Health Research and Development in 2018. Data collection takes place from 2018 until 2022. Data analysis starts after the last round of data collection in 2022 and finalizes in 2024. Expected results will be published in 2023 and 2024.

**Conclusions:**

Despite the societal relevance of SCEs, little empirical research has been performed on their functioning and impact. This research applies a variety of methods and includes the perspectives of multiple stakeholders aiming to generate new empirical evidence. The results will enable us to describe how SCE activities influence intermediate and long-term health outcomes and how the organizational and sociopolitical context of SCEs may shape opportunities or barriers for health promotion. As the number of these initiatives in the Netherlands is increasing rapidly, this research can benefit many SCEs attempting to become more effective and increase their impact. The findings of this research will be shared directly with relevant stakeholders through local and national meetings and annual reports and disseminated among other researchers through scientific publications.

**International Registered Report Identifier (IRRID):**

DERR1-10.2196/37966

## Introduction

### Background

Despite many efforts, national policy in the Netherlands has not been able to affect the persistence of health inequalities. The health of people with a low socioeconomic status (SES) has always lagged far behind that of people with a high SES [1]. Life expectancy of people with a low SES is 6 to 7 years lower than that of people with a high SES, and the difference in healthy life expectancy is even greater, namely 14 years [2]. In the Netherlands, socially vulnerable groups, including those with low SES, are generally less healthy and less engaged in health-promoting activities than higher SES groups [3]. As in many countries, health in the Netherlands is also unevenly distributed across residential areas [4,5]. An effective method in reducing health inequalities may therefore lie in a process-oriented neighborhood development approach [6,7]. Social community enterprises (SCEs) offer such an approach in which residents of disadvantaged neighborhoods can participate in society, be stimulated to live healthier lives, and play an active role in their own community’s development. Examples of SCEs are organizations that run a small laundry facility in the neighborhood for vulnerable families, promote work activities for new immigrants, or organize cultural and creative activities in a poor district. It is crucial for a social enterprise organization that its objectives are primarily social, and that its surpluses, arising from revenues of commercial activities, are principally reinvested to achieve these social objectives [8]. If a large proportion of the participants come from the surrounding district in which the organization is located and its activities are strongly directed toward the development of the district and its residents, that organization is considered an SCE [9].

SCEs have been linked to various beneficial outcomes, at both individual and community levels. For instance, SCEs are expected to provide a cheaper alternative to costly governmental urban development and might contribute to safety and livability of the neighborhood [10], employment opportunities for excluded groups [11], and social inclusiveness [12]. In addition, Roy et al [13] found evidence that “social enterprise activity can impact positively on mental health, self-reliance/esteem and health behaviors, reduce stigmatization, and build social capital.” However, past research has delivered limited evidence of the benefits of SCEs, and empirical studies on how and to what extent they can contribute to health and well-being are rare. Research on beneficiaries, such as participating residents, is similarly scarce [13].

It remains unclear how and to what degree the activities of SCEs impact the health and well-being of residents in vulnerable districts. Therefore, as a first goal, this paper outlines a protocol for an evaluation study aimed at gaining more insights into the health outcomes of SCEs at individual and community levels, specifically investigating the extent to which SCEs affect the health and well-being of participating residents.

Besides outcomes, surprisingly little empirical research has been done on the underlying processes and mechanisms of health impact [14,15]. Thus, it remains unclear how involvement in the activities of SCEs might lead to improved health outcomes. It is known that many SCEs organize social as well as commercial activities on a neighborhood level. What this research aims to clarify is how participating in these activities might strengthen people’s health. Possibly important factors here are an increase in self-esteem, the prospective of having weekly social activities such as a weekly lunch or walking exercise, or a sense of belonging and ownership.

SCEs seem to share some common features with social care farms and green citizen initiatives, such as an orientation toward empowerment, strengthening of assets, and a focus on communities [16-18]. Care farms combine agricultural production with health, social, and educational services, like the provision of day care, supported workplaces, and residential places for clients with a variety of disabilities [19-21]. Green citizen initiatives constitute urban-based services such as community and institutional gardens or city farms. In particular, social care farms entail a shift in care in recent decades characterized by the terms deinstitutionalization, socialization, and normalization and a shift from institutional to community care. Studies based on the experiences of social care farms and green citizen initiatives indicate that, for a variety of citizens with specific needs, the key to improving the quality of life of participants in SCEs lies in meaningful and activating activities, a safe and welcoming community, and an informal context that is close to normal life [16,22]. Thus, it is important to understand the interplay that takes place between participation in SCE activities and health development and to create insight into the processes and mechanisms that underlie it. Therefore, the second goal of the evaluation study is to gain more insights into the processes and mechanisms that are at work in these SCEs and that determine the impact of the residents’ participation on outcomes of health and well-being.

Whether an SCE has impact on the health and well-being of residents is also determined by the organizational and sociopolitical context in which these initiatives operate [13]. One crucial condition is that this context can create opportunities for the SCE initiatives to thrive and strengthen the assets of individuals and communities [12]. Context concerns factors such as the capabilities of initiators; their organizational form; legal setup; number of activities and projects; management style of the organization; district in which SCEs operate and communities that are linked to them; networks of boards; funding from government officials and commercial, social, or cultural organizations; and collaboration with such institutions. For example, De Bruin et al [23] state that care farming organizations, which combine commercial and social activities in a similar way to SCEs, require an empathic, creative, innovative staff that knows how to align meaningful activities with personal needs, support a sense of mastery, and facilitate engagement of participants.

These factors might be decisive for the extent to which SCEs can succeed at improving the well-being of residents and the livability of districts. This success has to do with their position in relation to other stakeholders in the context of the market, and of the local, regional, and national government. One crucial contextual factor might be the capacity of these initiatives to create collaboration with governments, nongovernmental organizations, and commercial parties that can provide the necessary conditions for sustaining and expanding their activities [12]. Another crucial consideration is whether efforts from the SCE in building collaboration with other stakeholders will also provide participants with opportunities to strengthen their assets. In that sense, it is relevant to investigate to what extent the SCEs use organizational strengths such as the capabilities of initiators and efforts of volunteers to successfully create conditions in their environment that lead to improved health and well-being of residents and communities. Hence, a third goal of the evaluation research is to explain how the potential of SCEs in strengthening individual and community assets is determined by organizational and contextual factors.

### Theoretical Framework

The theoretical basis of the evaluation research can be found in an asset-based model of health and 2 conceptual models, namely the social enterprise intervention model by Roy et al [13] and the empirically informed conceptual model by Macauley et al [24]. On the basis of these models, we have constructed an expanded model that functions as the theoretical framework of this research.

### Asset-Based Model of Health

The asset-based model of health emphasizes the capabilities of persons and opportunities for collaboration in communities and organizations to sustain and promote health [25,26]. The approach is based on the salutogenic model of health [27], which means that by focusing on assets instead of problems or deficiencies, it is possible to identify factors and mechanisms that allow people to move toward the health end of the spectrum between ill health and health. The fundamental premise is that individuals will do better in the long run if they are supported to identify, recognize, and use the strengths and resources available in themselves and their environment [28,29]. On a community level, asset approaches can help people to discern and use those skills, resources, knowledge, and connections within communities that can promote health and support well-being [30]. For instance, social enterprises can be effective in providing employment opportunities and creating more enterprising communities [11]. By strengthening residents’ assets, SCEs can contribute to social cohesion and improve their quality of life, health, and well-being [12]. Moreover, low-income residents involved in these community initiatives can accrue 4 different nonfinancial assets (ie, social, cultural, human, and political capital) that can improve their health and well-being [25,31]. This is an iterative process in which residents’ improved health and well-being further support the acquisition and development of new assets.

According to Benenson and Stagg [31], SCEs may call on the assets that are already available in the community as well as enable the development of new assets on both individual and community levels. The activities of the SCEs aim to strengthen the capacities of residents to participate in society—for example, by offering skill lessons for getting a job or by developing additional social relationships to reduce loneliness. On a community level, these enterprises may seek to support community health by creating a green and safe physical environment and by increasing social cohesion. We expect that increased availability and use of individual and community assets will support residents and communities in dealing with the challenges they face, thus strengthening their health and well-being. For example, by sharing experiences on health issues in familiar settings, participants may strengthen their health literacy.

### Integrated Model of SCE Health Intervention

**Figure 1:**
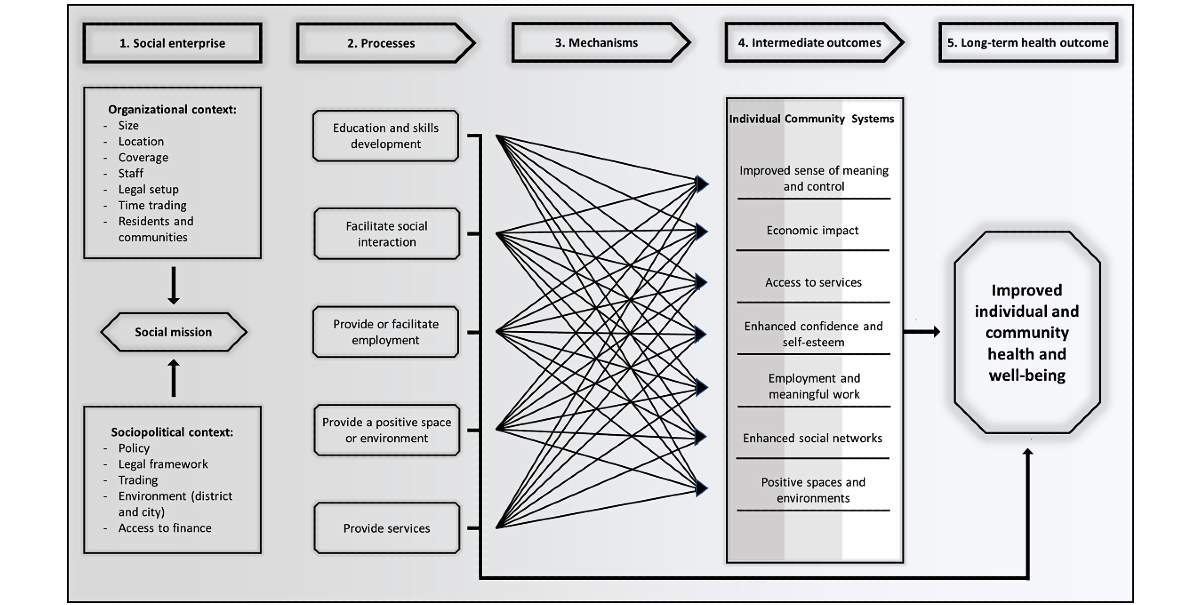
Integrated model of the social community enterprise health intervention based on Roy et al [13] and Macaulay et al [24].

The theoretical framework is further based on the integration of 2 conceptual models developed to strengthen our understanding of how SCEs can contribute to health outcomes. Both aim to describe how activities by SCEs can impact intermediate and long-term health outcomes. The first is the model by Roy et al [13], and the second is the model by Macauley et al [24]. To fit our research questions, several adaptations have been made to these models to create an integrated model (see Figure 1).

The model by Roy et al [13] puts forward a chain of causality containing the different steps through which intermediate and long-term health outcomes are generated. These steps involve the (1) internal and external factors determining the social mission of a social enterprise, (2) intervention, (3) intermediate effects, and (4) long-term outcome. The assets include emotional well-being, social networks and relationships, good work, and social functioning. The long-term outcomes revolve primarily around social capital, connectedness, and sense of coherence, leading to improved health and well-being. The elements describing the factors that determine the social mission of the SCEs are necessary to answer our second research question on processes and mechanisms and our third question on assets. From this model, we have reframed the factors determining the social mission as the organizational and sociopolitical context.

In their model, Macauley et al [24] elaborate in more detail on the long-term health outcomes on which SCEs might have an impact. In this model, these are improved sense of meaning and control; economic impact; access to services; enhanced confidence and self-esteem; employment, employability, and meaningful work; enhanced social networks; and positive spaces and environments. In line with the assets model by Morgan and Ziglio [26], the health outcomes of the model by Macauley et al [24] can take place on 3 levels: individual, community, and system. Thus, the model describes the impact exerted on the different levels by processes (ie, activities that, intentionally or not, may lead to positive health outcomes) and mechanisms which form chains of causality leading to better situations of health and well-being.

As a final adaptation, we have strengthened the aspect of communities and the sociopolitical context of the district and the city in our model (see under social enterprise, Figure 1). This also relates to a wider development in the Netherlands: the emergence of a stronger district-oriented approach by local government in the last decade, which has had an especially notable impact on the opportunities for neighborhood initiatives within the sociopolitical context in Dutch municipalities, and the rise of community-oriented social enterprises [32]. For example, SCEs that are embedded within specific districts might have the advantage that they can stimulate participation of residents in such communities more than organizations oriented on the level of an entire city. One of the reasons for a potentially higher impact is that these individuals might be more motivated by the fact that their efforts benefit the neighborhoods in which they themselves live. The integrated model combining the relevant analytical elements for our research is presented in Figure 1.

To conclude, the study outlined in this protocol aims to contribute knowledge on the potential of SCEs to impact health and well-being and reduce health inequalities. The following research questions were formulated:

- What is the impact of SCEs on health outcomes at individual and community levels?
- What underlying processes and mechanisms can explain the possible health impact of SCEs?
- What assets are available to SCEs through their organization and their context, and how can SCEs successfully mobilize these assets?

## Methods

### Design

This research applies a mixed methods multiple-case study design including repeated cross-sectional measurements. The cases to be studied are 6 SCEs located across 5 vulnerable neighborhoods in a medium-sized Dutch city. We will use questionnaires, interviews, and observations as research instruments.

### Setting

Before the start of this study, interviews will be held with the 6 selected SCEs to ensure that they conform to the definition stated earlier. All SCEs focus to a large degree on improving the district they are located in. They all operate in districts that face a combination of serious problems such as low average levels of physical and mental health, high levels of unemployment, low levels of participation and education, low levels of social cohesion and livability, and perceptions of deterioration and lack of safety of the neighborhood. Every community enterprise in our study aims to reduce these problems: that goal is the fundamental reason for the existence of the enterprise. A core principle these SCEs share is that they are convinced that the social problems should be dealt with through the participation and self-management of the district’s residents. The community enterprises take the residents’ assets as a starting point and develop activities from there onward. Other fundamental principles include taking an entrepreneurial attitude by the initiators and promoting such an attitude among their participants (eg, by creating a cooperative of small businesses).

The selected SCEs differ, however, in context, in the demographic profile of the districts and participants, in the differences in target groups, in the main problems of the residents and the districts, and in the type of entrepreneurs and activities. Some of these initiatives attract only vulnerable residents such as unemployed persons and asylum seekers, while others organize activities that are directed at all residents and attract persons with both low and high SES. Examples of activities are cultural activities for people to meet each other, activities that improve the neighborhood such as greening of public spaces and garden maintenance, and strictly commercial activities such as managing parking lots or a small bicycle shop. Depending on the type of activities, these SCEs attract from a dozen to a hundred persons on a weekly basis. The diversity of these social enterprises allows for a cross-case comparison demonstrating which processes and mechanisms apply to which contexts, and which types of asset building lead to which effects.

### Data Collection

#### Health Impact on Individual and Community Levels

To evaluate the impact of the 6 SCEs on health and well-being, quantitative data will be collected from participants in the activities of the selected SCEs during the duration of the project. Data collection, including administering questionnaires and holding interviews, will take place at the locations of the SCEs. The anonymity of the participants will be safeguarded by several measures such as the use of separate rooms where residents can be interviewed and complete the questionnaires in private. The procedure for the selection and recruitment of the participants will be coordinated in advance with the initiators. In our study, participants of the SCEs will be recruited randomly by the researchers, except for those residents who the initiators believe might find participation too burdensome. Both participants and the initiators from the SCEs will be notified beforehand about the aim of the questionnaire, the main topics, and the anonymous way in which the information will be used, and their consent will be requested. Residents who start an activity in year 1 of our research will receive follow-up questionnaires for 3 years; residents who start in year 2 are followed for 2 years, and so on. This approach is expected to result in a sample of 270 participants across the 6 community enterprises (ie, 45 participants per neighborhood). Considerable effort will be put into encouraging residents who stop participating in the activities of the community enterprises to continue completing the questionnaires until the end of the project.

The outcome measures concern the intermediate health outcomes as presented in the integrated model (see Figure 1). On an individual level, these are sense of meaning and control; confidence and self-esteem; employment, employability, and meaningful work; and physical health. Physical health will be added as an outcome to the model, as the initiatives directly and indirectly influence health literacy by offering healthy lunches and social participation in sports activities. On the community level, the outcome measures are economic impact, access to services, social networks, and social cohesion. Several existing or validated instruments will be used as input for the questionnaire (see Table 1). Examples are the University of California, Los Angeles Loneliness Scale to measure social connectedness and the Dutch General Self-Efficacy Scale and Dutch Rosenberg Self-Esteem Scale to measure confidence and self-esteem. Table 1 describes which outcome measures are part of the questionnaire and the original instruments from which the questions were derived.

The questionnaires will be administered on paper or online. SPSS (version 25.0, IBM Corp) will be used for descriptive statistics for every measurement; the follow-up measurements will examine developments of participants. The data will be analyzed using multilevel regression models in SPSS and SAS (SAS Institute Inc) statistical software.

**Table 1:** References related to the questionnaire outcome measures.

Outcome measure	Original instrument
Educational level	Municipal Report Livability and Safety in the Neighborhood 2017 [33] GGD^a^ Monitor Gelderland-Midden [34]
Social connectedness	UCLA^b^ Loneliness Scale–CBS^c^ [35]
Living environment	Municipal Report Livability and Safety in the Neighborhood 2017 [33]
Sense of meaning and control	Adjusted version of the Daily Meaning Scale [36]
Confidence and self-esteem	Dutch General Self-efficacy Scale–Short form [37-39] Dutch Rosenberg Self-Esteem Scale [40]
Resilience	GGD Monitor Gelderland-Midden [41]
Overall health	GGD Monitor Gelderland-Midden [34] PROMIS^d^ Scale v1.2–Global Physical Health G03 [42,43]
Economic impact	GGD Monitor Gelderland-Midden [34]
Self-perceived impact of participation at the SCE^e^	The Work and Meaning Inventory [44]

^a^GGD: Municipal Health Services (Gemeentelijke Gezondheidsdienst).

^b^UCLA: University of California, Los Angeles.

^c^CBS: Central Bureau for Statistics (Centraal Bureau voor de Statistiek).

^d^PROMIS: Patient-Reported Outcomes Measurement Information System.

^e^SCE: social community enterprise.

#### Underlying Processes and Mechanisms on the Individual Level

To understand the underlying processes and mechanisms on the individual level, interviews will be held with 2 groups. To gain a better understanding of the assets that are mobilized through community enterprises’ activities, in-depth qualitative interviews will be performed with participants in those activities. Each year, 4 to 5 participants per SCE will be invited to take part, resulting in a total sample of 16 to 20 participants over 4 years for each neighborhood. The interviews will be held with people who have been involved in the activities for a longer period of time and who are also participating in the questionnaire research. The interviews will be conducted by members of the research team. Each interview will follow a predefined semistructured format. This will ensure that the retrospective interviews focus on understanding which individual and community assets are mobilized through the participation in the activities and how the mobilized assets lead to better health.

Participants and the initiators from the SCEs will be notified beforehand about the aim of the interview, the main topics, and the anonymous way in which the data will be used. At the start of the interviews, permission will be requested to record the conversation. All recordings will be transcribed, and both audio files and transcriptions will be stored at a secure site. The transcripts will be analyzed by thematic coding and content analysis using Atlas ti.8 (Scientific Software Development GmbH). This analysis will be directed at unravelling the mechanisms of change on an individual level. Quotes that reveal essential elements of the processes, mechanisms, and outcomes at stake will be selected to illustrate our findings. Furthermore, semistructured in-depth interviews with the initiators of the SCEs, district managers, and social district team employees will be conducted each year.

#### Underlying Processes and Mechanisms at the Organizational Level

Information at the organizational level will be collected by interviewing initiators of SCEs, social district team employees, and the district managers of the municipality. At least 3 persons per initiative per year will be interviewed, which will add up to a minimum of 45 interviews. Semistructured in-depth interviews will be scheduled during the first, second, and fourth year of the project, at time points to be determined, to be able to document changes in the approach of the community enterprises. The interviews will focus on the community and organizational assets mobilized through the SCEs. In addition, they will inquire about the constraining and facilitating factors in the collaboration between the community enterprises and other stakeholders, such as the local government. Besides that, the competencies and activities of the initiators, as well as their expectations, wishes, and experiences, will be explored. The interview questions will concern (1) changes in their approach and activities, (2) the participation and involvement of residents, (3) the assets of participants as individuals and groups, and (4) the initiatives themselves. The data from these interviews will allow us to identify the factors that play a role in the success of SCEs and the implementation of their activities in local policies. With these insights, the approaches of the enterprises and the policies of the municipality can be improved.

During the 4 years of the research project, observations per initiative will be made on the mechanisms of change on the organizational level through participatory research (ie, making notes during informal happenings) and from informal communication (eg, email conversation, phone calls) with the initiators. Using analytical schemes, structured observations will be collected in which we will describe the approach of the staff of the initiatives and their concrete actions and opinions, interactions with participants and stakeholders and their actions and opinions, and the processes and mechanisms described earlier. Comparisons between the SCEs and their organizational and political settings will be made. These insights will provide us with an improved understanding of success or failure of SCEs and their different approaches.

### Data Triangulation and Analysis

By applying a variety of research methods, this study aims to assure the validity of this research and make it possible to examine different dimensions of the phenomenon of SCEs. Data from the questionnaires and interviews with participants, initiatives, and stakeholders will be combined with our own observations. This data will provide insights into the context, processes, and mechanisms at work that form potential pathways along which assets are strengthened and participants at SCEs can gain improved health. In particular, the insights into mechanisms that explain how participants’ behavior is determined by their involvement in these community enterprises will make it possible to evaluate the complex components of approaches that target health improvement in such settings. In this way, elements such as the relationship with the initiator or the involvement in a local community can be identified as determining factors. By focusing not on projects but on the processes and mechanisms that form different pathways in varying contexts, our study can gain insights that are applicable to other settings.

This study will use data extracted from stakeholders to incorporate different perspectives on improvement of health into our analysis. Via methods such as interviews, questionnaires, and observations, insights can be questioned and tested to see if they support or contradict patterns derived from the separate research instruments. Information from the interviews with participants, initiators, and stakeholders can lead to the identification of mechanisms. The collected quantitative data can then be used to question and test these identified mechanisms.

### Ethical Approval

Participants and initiators from the SCEs will be notified beforehand about the aim of the questionnaire, the main topics, and the anonymous way in which the information will be used. All participants will be asked to provide permission via a written consent form. It will be made clear to participants that participation is voluntary and withdrawal from the study is possible at any time for any reason. We will monitor the number of persons who do not want to take part in this study and will record their reasons for not participating. The data collected will be treated confidentially and pseudonymously, which means that identifiable elements will be collected separately and will be encoded. This will ensure that the data cannot be traced back to any of the participants. The data set will be encrypted and stored in a repository with restricted access. This research will be conducted in compliance with the ethical rules for social science research. We have acquired approval for this study from the Wageningen Social Sciences Ethics Committee (CoC number 09215846).

## Results

This research project received funding from The Netherlands Organization for Health Research and Development in 2018. Data collection takes place from 2018 until 2022. Data analysis will start after the last round of data collection in 2022 and will be finalized in 2024. Expected results are to be published in 2023 and 2024.

## Discussion

### Scientific Relevance

Despite the societal relevance of SCEs, little empirical research has been performed on their functioning and impact [14,15,45]. As Roy et al [13] suggested, this protocol article describes an evaluation study whose aim is “to better understand and evidence causal mechanisms and to explore the impact of social enterprise activity, and wider civil society actors, upon a range of intermediate and long-term public health outcomes.” The findings of this research can generate new empirical evidence on the health impact of SCEs and relevant processes, mechanisms, and organizational and sociopolitical contexts. With our results, we will be able to describe in more detail how the activities of SCEs can impact intermediate and long-term health outcomes and clarify the interplay between participation and health through the activities at these initiatives. More specifically, our research can contribute to the substantiation and further refinement of the conceptual model, as we already aimed to do in the integrated model of SCE health intervention presented in Figure 1.

### Societal Relevance

Many policy makers deal with questions regarding the added health value of community enterprises for vulnerable residents and deprived communities [14,45]. In turn, many SCEs struggle when trying to clarify the impact they can have on residents and communities. As the number of community enterprises in the Netherlands is increasing rapidly, this research can be beneficial for many initiatives attempting to become more effective and increase their impact among residents in deprived neighborhoods by strengthening the assets of their organizations, participants, and districts. Next to improving SCEs, our research can provide more traditional welfare city-based organizations with insights on how to promote health via the context of district-based communities.

### Strengths and Limitations

This study will follow 6 initiatives extensively during a prolonged period. These 6 SCEs can be described as diverse, yet they share a number of common principles. Therefore, during the research period, the research team will be able to study a wide range of settings and situations, providing the opportunity to study the impact of different approaches on health and well-being outcomes. We will follow the 6 initiatives throughout a period of 4 years. After each year, we will offer SCE professionals a report of the research results so that they can learn directly from the study. The SCEs will benefit from this research by learning from these insights and sharing their experiences, approaches, and methods with each other. In addition, in the third and fourth year of the research period, preliminary results will be shared on local, regional, and national levels with other SCEs and local and regional governments. Another strong point of this research is its mixed methods design. When different methods for measuring the same processes and mechanisms result in the same outcomes, this is extra support for our findings.

This study faces several challenges. First, to collect the data as described (ie, interviews, questionnaires and observations), this research will be dependent on the cooperation of many parties, namely the participants, initiators, and stakeholders such as the district managers and social district employees. This is a challenge that we aim to overcome by investing strongly in the relationships with the initiators and other stakeholders, even before the start of the research project. Regular meetings will be scheduled with the initiators during the project to maintain trusting and constructive relationships that provide a support base for this research.

A second challenge is that the number of respondents that can be recruited is dependent on possible growth or downsizing of the selected initiatives during the research period. At least 3 initiatives are limited in size and have been established recently. Hence, we must take into account that during the research period these initiatives might collapse, leaving us with limited collected data. In addition, an initiative may change its approach drastically; for example, it might cease to aim its activities at the neighborhood or at residents with low SES. We have taken this risk into account by selecting more initiatives than strictly necessary, which will make it possible to reach the required number of participants for this research even if one of the initiatives withdraws from the project.

Last, as many other factors can determine possible positive effects on the health of the participants of the SCEs, this research cannot deliver hard evidence for any causal relations between their health and their participation in activities at the SCEs. Likewise, this research will not entail control groups in other districts among different types of organizations. However, by using in-depth interviews, structured questionnaires, and observations, this research can apply data triangulation, which will make it possible to gain more insights into the causal relationships that determine the health outcomes at SCEs.

In addition to its internal validity, this research will also need to be externally valid. The fact that this research is limited to one municipality will restrict the extent to which the conclusions can be generalized to other situations. This problem will be partly overcome by the use of multiple, diverse cases. Finding comparable processes and mechanisms in these different settings will help to provide a basis for generalizing the results to comparable situations for SCEs in other sociopolitical contexts [46].

### Valorization and Dissemination Plan

National and local governments can benefit from this research, as we provide insights into beneficial forms of collaboration between initiatives and government. In this way, this study can provide input to improve policy. In response to the inequalities mentioned above, it is Dutch policy to promote community-based health-enhancing programs that improve the health and well-being of socially vulnerable groups [47]. These programs emphasize intersectoral collaboration and build on concepts like supportive environments, community participation, and community ownership [3]. In line with this, there is a growing interest within national and local governments in involving residents in district-oriented entrepreneurial activities. The Dutch government has published a white paper that emphasizes its aim of supporting residents in taking up societal issues [48]. One way for residents to do so is to participate in an SCE.

Insights into the mechanisms of how SCEs possibly improve residents’ health and well-being can make local policy and programs more effective. To promote our research results and recommendations for SCEs and local governments, we will organize local and national meetings and workshops at which SCEs and similar initiatives can exchange thoughts and findings with policy makers and other stakeholders. Furthermore, we will collaborate with expert organizations, such as the Provincial Alliance on Livability, Pharos (Dutch center for expertise on health inequalities) and Movisie (Dutch knowledge institute for social issues). Their role in this collaboration is twofold: to deliver expertise and offer us opportunities to discuss our results with other SCEs and local decision makers and policy advisors in the Netherlands.

The results of this research will be shared with other academics through publication in international open-access peer-reviewed journals. The quantitative data of this research project will be made available on request via the restricted access functionality in Data Archiving and Networked Services–Electronic Archiving System (DANS-EASY) after an embargo period to allow publication of results (maximum 2 years, conforms with DANS-EASY embargo period). The qualitative data are not open access but will be available on request.

## Appendix

Multimedia Appendix 1 Review 1 decision project 531001317 by the Nederlandse organisatie voor gezondheidsonderzoek en zorginnovatie (ZonMw, The Netherlands Organisation for Health Research and Development) - Preventieprogramma 5 (Prevention Program 5).

Multimedia Appendix 2 Review 2 decision project 531001317 by the Nederlandse organisatie voor gezondheidsonderzoek en zorginnovatie (ZonMw, The Netherlands Organisation for Health Research and Development) - Preventieprogramma 5 (Prevention Program 5).

Multimedia Appendix 3 Review 3 decision project 531001317 by the Nederlandse organisatie voor gezondheidsonderzoek en zorginnovatie (ZonMw, The Netherlands Organisation for Health Research and Development) - Preventieprogramma 5 (Prevention Program 5).

## Abbreviations

DANS-EASY: Data Archiving and Networked Services–Electronic Archiving System
SCE: social community enterprise
SES: socioeconomic status
