# Supplementary material for: The Health Impact of Social Community Enterprises in Vulnerable Neighborhoods: Protocol for a Mixed Methods Study
Source: JMIR Res Protoc. 2022 Jun 22;11(6):e37966. doi: 10.2196/37966 (PMC9260530; doi:10.2196/37966)
Supplement: Multimedia Appendix 1 [file resprot_v11i6e37966_app1.pdf]

Subsidieprogramma / Subsidy programme	:	**Preventieprogramma 5**
Dossiernummer / Dossier number	:	**50-53100-98-208**
Aanvrager / applicant	:	**Drs. E. Hendriks**
Projecttitel / Project title	:	**Addressing health inequalities in vulnerable urban neighbourhoods by social community enterprises Sociaal economische gezondheidsverschillen in kwetsbare wijken verkleinen door Sociale Wijkondernemingen**
Beoordelingscode / Assessment code	:	**B.2017.014F7**

1. Criteria

Legenda: E (Excellent), G (Good), S (Sufficient), M (Moderate), U (Unsatisfactory)

1.1 Objective, problem definition and assignment

E	G	S	M	U
	X			

Consider the following factors:

- the objective is clear and specific . The overall aim is realising health effects by focusing on multiple determinants/factors;
- the problem definition/assignment is clear and verifiable and is consistent with the objective;
- the value added to existing knowledge or practice;
- the theoretical or empirical evidence presented in support of the problem definition/assignment.

The project uses a very "broad" perspective on health, without clearly specifying what dimensions of health will be monitored or assessed (cfr infra)

1.2 Strategy

E	G	S	M	U
	X			

Consider the following factors:

Research strategy
- clarity;
- adequacy in terms of problem definition/assignment;
- adequacy of chosen methods and analyses;
- adequate inclusion prospectives of the target group
- the target probative value should be at least 'initial indications of effectiveness';
- the way in which the strategy reflects the factors gender, age, ethnicity and socio-economic health inequalities;
- degree of collaboration with intermediate and ultimate target group (the client perspective);
- it may be that the scientific study of the effect of the intervention is at odds with the monitoring of integrated policy programmes. This should be mentioned explicitly, and the choices which are made as a result should be clear.

Implementation strategy
- analysis of the context and community in which implementation is to take place;
- extent to which target groups are mentioned;
- the integrated approach (sum total of interventions) should be clearly described, even if it is not yet fully developed;
- analysis of factors facilitating or hampering those activities;
- local authorities are playing an increasingly important role in the development of integrated health policies;
- prospect of structural incorporation in system;
- adequacy of process and effect evaluation design.

While selecting 4 "living lab" settings in 4 neaighbourhoods is cetrainly defendebale, it is not entirely clear how the "precise" social mix of particpants will afeect the organisation and the needs of the participants. Whilde the sociale participation activation and developing self initiative is a valuable objective, one should take into account that the level of selforganisation and the competencies and capacities might differe greatly between audiences. Moreover, form a reserach point of view this makes the assement to what extent the initiave relyy contributes to health chages particularly challenging.
In my point of view the proposal is a bit "normative" in the sense that it not very attentive to the possible strain or netgative stress these type of initiatieve might cuase for particular types of participants. This part should be considered a bit more explicitelty in order to be able to work towards sustainability. especially this latter aspect is

not strongly elaborated, and nether is strongly elaborated on how the evaluation part will contrute to develop sustainable strategies.

1.3 Project group

E	G	S	M	U
	X			

Consider the following factors:

- relevant expertise;
- familiarity with area in question;
- prior activities and products.

The team is clearly acquainted with the approach/methodology and obejective of the proposed intervention, although we need to mention that the "concrete" methods used to empower and enhance activities and health of a vulnarable population is not elaborate din the proposal. The team seems less acquainted with the comprehension ans assment of the "health" dimension, which is a core focus of the programme.
The mix of expertise and the embeddedness within the city are strong indicators for potential succes of this project. However, if the p^roposal is honoured, the team should pay sufficitant attention to the methodlogical basis of the prospective evaluation and how the lessons learned will contribute to the adaptaion and changes in the construction of the living lab experience, particilalr form a health perspective.

1.4 Feasibility

E	G	S	M	U
	X			

Consider the following factors:

- will it be possible to achieve the objective(s) using this strategy?
- availability of facilities/staff;
- realistic phasing and timetable;
- analysis of factors which may positively or negatively impact the feasibility;
- feasibility of the collaboration with relevant stakeholders and intermediate target groups.

Considering the broad and very lilltje elborated description of the "health concept" it might be difficult te really assess the objective(s) to measure impact. Moreover, considering the wide variety of interventions within the living lab setting, it will be very difficult to clearly assess whether the strategy aims at improving health of the probably wide variety of participants
The timing could be realistic to implemnt the methdologyanalysis of factors which may positively or negatively impact the feasibility;
feasibility of the collaboration with relevant stakeholders and intermediate target groups.

1.5 Overall quality assessment

E	G	S	M	U
	X			

This is a typre of intervention that fits within the "integrated" and participatory approches which are particlarly put forward to integrted the weakest soci-econimic and culturel groups in society.

The operationalisation of the health dimension is not clear, and the use of the widest interpretation of the term might and will blur the interpretation of the impact of the intervention.

I really would recommend to equally foucs on process eveluation and implention reserach on barriers and facilitating factors for reaching the target poulation. There will be a real risk that the settings will reacht vulnarable people but not really the "weakest" vulnarble groups for which these approaches are generally intended

The starting point and underlying assumptions about developing demonstrated effects are slightly naive and optimistic. There is a real risk in using the "broadest health concept" while the intervention and living lab model is actually focussing on particiaption and empowerment. The proposal has not elaborated on how the baseline health conditions of participant will be taken into account and how the "health changes" will be measured. The propesed reserach stratgey coul lack sensitivity and potentially also validity.
But overall if a true process evaluation and a well organised monitoring freamework is developed , these type of intiatives will develop valuable knowledge to test innovative social innovation stretegies and roll out future and sustainable policy for vulnarable populations

2. Budget

Legenda: TH (Too high), R (realistic), TL (too low)

2.1 Budget

TH	R	TL
	X	

This budget is realistic. one slight concern is that for these types of interventions a lot of human suppoprt will be continuousle needed. It slightly concernes me that a vast amount of the budget is demanded for "material cost" compared to personnel, and that no co-financing is foreseen.

The proposal is not really explaining what resoeruces are needed for what part or WP in the role out of the activities
