# Supplementary material for: The Health Impact of Social Community Enterprises in Vulnerable Neighborhoods: Protocol for a Mixed Methods Study
Source: JMIR Res Protoc. 2022 Jun 22;11(6):e37966. doi: 10.2196/37966 (PMC9260530; doi:10.2196/37966)
Supplement: Multimedia Appendix 2 [file resprot_v11i6e37966_app2.pdf]

Subsidieprogramma / Subsidy programme	:	**Preventieprogramma 5**
Dossiernummer / Dossier number	:	**50-53100-98-208**
Aanvrager / applicant	:	**Drs. E. Hendriks**
Projecttitel / Project title	:	**Addressing health inequalities in vulnerable urban neighbourhoods by social community enterprises Sociaal economische gezondheidsverschillen in kwetsbare wijken verkleinen door Sociale Wijkondernemingen**
Beoordelingscode / Assessment code	:	**B.2017.014F8**

1. Criteria

Legenda: E (Excellent), G (Good), S (Sufficient), M (Moderate), U (Unsatisfactory)

1.1 Objective, problem definition and assignment

E	G	S	M	U
X				

Consider the following factors:

- the objective is clear and specific . The overall aim is realising health effects by focusing on multiple determinants/factors;
- the problem definition/assignment is clear and verifiable and is consistent with the objective;
- the value added to existing knowledge or practice;
- the theoretical or empirical evidence presented in support of the problem definition/assignment.

The proposed approach and methods are broadly appropriate, and align with activities at local and regional levels in many western countries over the past ten years. The need for action and its significance is made, and this generally aligns with the research on the topic.

The emphases on (i) co-creation to mobilise existing networks and assets, and (ii) a broad intersectoral team are the most innovative aspect of this proposal, but the mechanisms by which this will occur could be described in greater detail.

The precise mechanism by which the community enterprises are theorised to lead to changes in social cohesion and safety is not clearly articulated, nor is how this will enhance health - but that is part of the justification for this research, and helps to explain the need for it. However these connections are described in the literature elsewhere.

The proposed translation of this project's activities and research findings is excellent. My suggestion is that in addition to transfer between sites and at the national level, that some consideration be given to dissemination internationally, as well as demonstrating the impact of research translation activities themselves. The findings will be relevant in many settings.

1.2 Strategy

E	G	S	M	U
	X			

Consider the following factors:

<u>Research strategy</u>

- clarity;
- adequacy in terms of problem definition/assignment;
- adequacy of chosen methods and analyses;
- adequate inclusion prospectives of the target group
- the target probative value should be at least 'initial indications of effectiveness';
- the way in which the strategy reflects the factors gender, age, ethnicity and socio-economic health inequalities;
- degree of collaboration with intermediate and ultimate target group (the client perspective);
- it may be that the scientific study of the effect of the intervention is at odds with the monitoring of integrated policy programmes. This should be mentioned explicitly, and the choices which are made as a result should be clear.

<u>Implementation strategy</u>

- analysis of the context and community in which implementation is to take place;
- extent to which target groups are mentioned;

- the integrated approach (sum total of interventions) should be clearly described, even if it is not yet fully developed;
- analysis of factors facilitating or hampering those activities;
- local authorities are playing an increasingly important role in the development of integrated health policies;
- prospect of structural incorporation in system;
- adequacy of process and effect evaluation design.

The research approach is broadly feasible and appropriately targeted. There may be challenges with attribution of findings, for example will changes (positive or negative) in social cohesion and safety be attributable to this project? The case study approach partially addresses these concerns, but they warrant further consideration.

A small concern is the relatively limited number of participants under research objective 1 activity 2 (questionnaires among participants). Will 60 per year over four years have sufficient statistical power to detect change? A component of the research design that is under-described is how will data on knowledge transfer itself (research objective 3) will be collected, analysed and reported.

The sites are appropriately selected with reference to “levelling up” approaches to addressing health inequalities, which recognise need and variation between them. The community assets that will be leveraged for the project are alluded to but not specified in great detail.

The Implementation Strategy seems sound, no specific comments.

1.3 Project group

E	G	S	M	U
	X			

Consider the following factors:

- relevant expertise;
- familiarity with area in question;
- prior activities and products.

The project group have good experience ad represent a diverse and appropriate team for the scope of the project. This project builds upon their prior related work between the social enterprises, government agencies, and the municipal government. The only apparent weakness is demonstrating the involvement of the researchers in activities with the project partners.

1.4 Feasibility

E	G	S	M	U
	X			

Consider the following factors:

- will it be possible to achieve the objective(s) using this strategy?
- availability of facilities/staff;
- realistic phasing and timetable;
- analysis of factors which may positively or negatively impact the feasibility;
- feasibility of the collaboration with relevant stakeholders and intermediate target groups.

Some aspects of the project will require more detailed planning (in particular research objectives 1 and 3), but the overall approach and team suggest the project is feasible. The scope of the project is realistic within the timeframes identified. The involvement of students is appropriate and will be beneficial.

1.5 Overall quality assessment

E	G	S	M	U
	X			

This is a useful project that has the capacity to make a contribution to local, national and international knowledge. The design addresses some of the potential challenges in attributing changes to the project, but more work may be required on this point. Further attention to investigating and demonstrating the impact of this project on policy and practice as part of its design will enhance its overall public value.

2. Budget

Legenda: TH (Too high), R (realistic), TL (too low)

2.1 Budget

TH	R	TL
		X

I think the budget is modest when considering the number of sites (4) and the scope of the research. The team have leveraged existing activities and the use of students, with considerable in-kind contributions. I think this risks

under-estimating the amount required for coordination of a complex research project and under-resourcing may pose a risk to the project overall.
