# Supplementary material for: The Health Impact of Social Community Enterprises in Vulnerable Neighborhoods: Protocol for a Mixed Methods Study
Source: JMIR Res Protoc. 2022 Jun 22;11(6):e37966. doi: 10.2196/37966 (PMC9260530; doi:10.2196/37966)
Supplement: Multimedia Appendix 3 [file resprot_v11i6e37966_app3.pdf]

Subsidieprogramma / Subsidy programme	:	**Preventieprogramma 5**
Dossiernummer / Dossier number	:	**50-53100-98-208**
Aanvrager / applicant	:	**Drs. E. Hendriks**
Projecttitel / Project title	:	**Addressing health inequalities in vulnerable urban neighbourhoods by social community enterprises Sociaal economische gezondheidsverschillen in kwetsbare wijken verkleinen door Sociale Wijkondernemingen**
Beoordelingscode / Assessment code	:	**B.2017.014F9**

1. Criteria

Legenda: E (Excellent), G (Good), S (Sufficient), M (Moderate), U (Unsatisfactory)

1.1 Objective, problem definition and assignment

E	G	S	M	U
		X		

Consider the following factors:

- the objective is clear and specific . The overall aim is realising health effects by focusing on multiple determinants/factors;
- the problem definition/assignment is clear and verifiable and is consistent with the objective;
- the value added to existing knowledge or practice;
- the theoretical or empirical evidence presented in support of the problem definition/assignment.

It is a project that tries to evaluate four social community enterprises. it is carried out in collaboration with the population affected and the local council of Arnhem.

A conceptual framework showing the relationship with the different determinants and health outcomes would help. Also showing how the intervention would affect outcomes.

The project has 3 main objectives and none of the 3 refers to health or inequalities in health (although they refer to health after in the text). From my point of view, it would be better if the researchers addressed health in the objectives.

1.2 Strategy

E	G	S	M	U
	X			

Consider the following factors:

Research strategy

- clarity;
- adequacy in terms of problem definition/assignment;
- adequacy of chosen methods and analyses;
- adequate inclusion prospectives of the target group
- the target probative value should be at least 'initial indications of effectiveness';
- the way in which the strategy reflects the factors gender, age, ethnicity and socio-economic health inequalities;
- degree of collaboration with intermediate and ultimate target group (the client perspective);
- it may be that the scientific study of the effect of the intervention is at odds with the monitoring of integrated policy programmes. This should be mentioned explicitly, and the choices which are made as a result should be clear.

Implementation strategy

- analysis of the context and community in which implementation is to take place;
- extent to which target groups are mentioned;
- the integrated approach (sum total of interventions) should be clearly described, even if it is not yet fully developed;
- analysis of factors facilitating or hampering those activities;
- local authorities are playing an increasingly important role in the development of integrated health policies;
- prospect of structural incorporation in system;
- adequacy of process and effect evaluation design.

The intervention is well explained and it seems to be already implemented in collaboration with the Local Council of Arnhem. It is implemented taking into account the population affected.

I have some concerns related with the evaluation methods:

- Quantitative approach:
a) it would be better to have a control group in order to compare the results with the groups with intervention.
b) health outcomes should be better explained.
c) It is not stated if the size of the sample is big enough.
d) Process evaluation should complement the impact evaluation proposed.

- Qualitative approach:
a) it is not explained how these data will be analysed.
b) A conceptual framework showing the main mediators would help.

- Axes of inequality should be better addressed. For example, gender should be taken into account.

1.3 Project group

E	G	S	M	U
		X		

Consider the following factors:

- relevant expertise;
- familiarity with area in question;
- prior activities and products.

The group of experts is not deeply explained. It is difficult for me to be able to know, for example, their expertise in health issues and in the implementation and evaluation of programs to reduce inequalities in health.

The expertise in working with communities is better justified.

1.4 Feasibility

E	G	S	M	U
	X			

Consider the following factors:

- will it be possible to achieve the objective(s) using this strategy?
- availability of facilities/staff;
- realistic phasing and timetable;
- analysis of factors which may positively or negatively impact the feasibility;
- feasibility of the collaboration with relevant stakeholders and intermediate target groups.

I think that the project can be carried out in 4 years because the four social community enterprises are already implemented and the project tries to evaluate them and to implement knowledge transfer.

Relevant stakeholders are taken into account.

The limitations of the project and the factors that could impact it should be better explained.

1.5 Overall quality assessment

E	G	S	M	U
		X		

I think that the project can be carried out, but the aspects I have mentioned above should be addressed.

2. Budget

Legenda: TH (Too high), R (realistic), TL (too low)

2.1 Budget

TH	R	TL
	X	

The budget seems realistic. Although it is not explained, I think that they ask for a researcher (4 years) and material to collect the information.
